# Health Tract, No. 45

**Published:** 1865

**Authors:** W. W. Hall

**Affiliations:** 42 Irving Place, New-York


### HEALTH TRACT, No. 45.

From "SLEEP," by Dr. W. W. Hall, 42 Irving Place, New-York.

### PRIVATE THINGS.

IF the urine is retained too long, the bladder becomes heated and inflamed, and loses its power; the inflammation then becomes more and more intense, and death takes place in two or three days. Hence, children should be taught to urinate the last thing before going to bed at night, and before leaving home for several hours, or going on a journey. The modesty of persons riding in stages has repeatedly resulted in death in this connection. As persons grow older, the call to urinate becomes more and more frequent. As early as fifty, it is necessary to arise several times during the night for that purpose; hence a vessel in the chamber of a guest is as indispensable as bed-clothing. The warmer the weather, the less the urine, and the more high colored, because so much of the water of the system escapes by perspiration through the pores of the skin; hence, they who labor most, urinate less than the sedentary. The color and quantity of urine depend so much on the greater or less amount of exercise, on the relative amount of food and drink, the quantity and quality of the latter, and the temperature of the weather, that none but a physician should draw conclusioas therefrom as to the state of the health. Hence, do not inspect the urine; and make it an imperative rule to give instant attention to a call. In males, attempts to urinate when the parts are turgid from any cause, rupture or stricture may result—a life-long calamity.

### STOOLING.

Every moment an even slight inclination to stool is resisted, the more watery particles begin to be absorbed into the blood again—a most filthy idea; and going on, that which is left behind becomes so dry and hard that it is impossible to void it, and the physician has to be called to spade it out with the handle of a spoon. The world-renowned surgeon, Dr. Valentine Mott, reports on one occasion having taken out eleven pounds from one individual. Costiveness is induced by deferring a call to stool to-day; to-morrow it comes later and later, until it occurs only two or three times a week. By this time health is impaired, piles are induced, falling of the bowels comes apace, so that whenever a passage occurs the pain is so insufferable, that it is necessary to lie down for several hours; or other ailments form, by which the excrements can not be controlled, and come away incessantly—a deplorable and disgusting condition!

These may be represented by cutting the rim of a purse, when the contents fall out of their own weight, often caused by straining too much, or remaining too long at stool, (five minutes are enough,) or by straining too suddenly when in a hurry. If a person finds, while on the privy-seat, that the excrements have begun to come, but there is reason to think that they are large and hard, it is infinitely best to introduce the finger carefully and take it out; there is nothing else you can do; a knife or stick would endanger wounding, while to strain on, would end in fissure.

The most consummate fools in nature are those who indulge outside of honorable wedlock, for lost self-respect and a blighted conscience to the end of life are inevitable results, while character is degraded, and in every case, even from a single fault, there is most imminent risk of a loathsome disease, which carries its baleful and degrading effects to generations yet unborn. The reflection is terrible. Self-indulgence brings on horrible bodily ailments, and destruction of the mind itself. See prevention and remedy in book above.

